# Antibacterial and Anti-biofilm Efficacy of Chinese Dragon’s Blood Against *Staphylococcus aureus* Isolated From Infected Wounds

**DOI:** 10.3389/fmicb.2021.672943

**Published:** 2021-06-04

**Authors:** Xiangkuo Zheng, Lijiang Chen, Weiliang Zeng, Wenli Liao, Zhongyong Wang, Xuebin Tian, Renchi Fang, Yao Sun, Tieli Zhou

**Affiliations:** ^1^Department of Clinical Laboratory, The First Affiliated Hospital of Wenzhou Medical University, Wenzhou, China; ^2^School of Laboratory Medicine and Life Sciences, Wenzhou Medical University, Wenzhou, China

**Keywords:** Chinese dragon’s blood, infected wounds, *Staphylococcus aureus*, antibacterial activity, anti-biofilm efficacy

## Abstract

Chinese dragon’s blood (CDB), a characteristic red resin, is an important traditional Chinese medicine (TCM), and empiric therapy of infected wounds with CDB is performed in clinical settings. For the first time, we herein report the antibacterial and anti-biofilm efficacy of CDB against *Staphylococcus aureus* (*S. aureus*). Antimicrobial susceptibility testing, growth curve assay, time-kill curve assay, crystal violet biofilm assay, scanning electron microscope (SEM) analysis, cell membrane tests, and quantitative real-time polymerase chain reaction (qRT-PCR) were used for this purpose. The results suggested that the minimum inhibitory concentration (MIC) values of CDB against *S. aureus* ranged from 32 to 128 μg/mL. Growth curves and time-kill curves confirmed that CDB could inhibit the growth of *S. aureus*. The biofilm formation ability and the expression levels of *saeR*, *saeS*, and *hla* of *S. aureus* in the presence and absence of CDB were statistically significant (*P* < 0.01). The results of SEM analysis and cell membrane tests revealed that exposure to CDB had some destructive effects on *S. aureus* cells. In conclusion, CDB exhibits positive antibacterial activity against *S. aureus*. Moreover, CDB could reduce the biofilm formation and the virulence factors of *S. aureus* by downregulating the expression levels of *saeR*, *saeS*, and *hla* genes. These findings indicated that CDB has immense potential to serve as a viable alternative for the treatment of infected wounds caused by *S. aureus* in clinical settings.

## Introduction

Worldwide, infected wounds are increasingly becoming a threat to human health (Ju et al., 2018). Acute wounds generally have a self-healing capacity and do not require significant external intervention; however, self-healing is often not possible in the case of chronic wounds. Therefore, external treatment is needed (Garcia-Villen et al., 2019). In fact, clinics face enormous challenges in managing chronic infected wounds. The normal recovery phases are altered significantly because of the presence of microbial contamination on the wound surface, leading to the possible impairment of the healing pathway and finally resulting in non-healing wounds (Garcia-Villen et al., 2019). Notably, *Staphylococcus aureus* (*S. aureus*) is frequently associated with infected wounds, and the pathogen is capable of biofilm formation (Salouti et al., 2016; Davis et al., 2017). Biofilms are adherent colonies of bacteria that are covered in a self-produced extracellular polysaccharide matrix (EPS) that is of host or mixed origin. Bacteria with biofilm phenotype undergo metabolic activity alterations within the protective EPS coating (Nair et al., 2016; Davis et al., 2017; Anderson et al., 2018). The biofilm enhances the ability of the organism to adapt to the environment, which in turn leads to reduced susceptibility to most antimicrobial agents. Some studies have established that alpha-toxin contributes to biofilm formation in *S. aureus* wound isolates (Anderson et al., 2012, 2018). Therefore, *S. aureus* biofilm formation plays a significant role in non-healing wound infections. The incorporation of antibiotics in treatment regimens has effectively eliminated multiple species of pathogens (Krychowiak et al., 2014). However, with the extensive use or even abuse of broad-spectrum antibacterial drugs, resistance to antimicrobial agents has been gradually increasing. The severe drug resistance status poses a huge challenge to anti-infective treatments in clinical settings (Song et al., 2016).

In this context, traditional Chinese medicine (TCM), including Chinese dragon’s blood (CDB), has been regarded as an alternative and complementary therapeutic intervention for infected wounds (Shen et al., 2019). According to the National Drug Standard [WS_3_-082 (Z-016)-99(Z)], CDB is a resin with rich, deep red color, which is obtained from the fat-containing wood of the lily tree belonging to the family *Liliaceae* (Wang et al., 2011, 2017; Lin et al., 2020). Preclinical studies have shown that CDB has many phytochemicals with anti-inflammatory, antimicrobial, antifungal, and antineoplastic properties and is therefore useful in the treatment of various diseases (Pona et al., 2019). As a natural remedy for infected wounds, CDB is widely used in treating sores, diabetic foot ulcers, soft tissue injuries, etc. (Ho et al., 2016; Pona et al., 2019). Therefore, our study aimed to investigate the antibacterial and anti-biofilm efficacy of CDB against *S. aureus* isolated from infected wounds. We further attempted to provide an experimental basis for the rational use of CDB for the treatment of infected wounds in clinical settings.

## Materials and Methods

### Clinical Isolates and Identification

A total of 46 *S. aureus* strains were isolated from the wound specimens of the patients from the First Affiliated Hospital of Wenzhou Medical University (Zhejiang Province, China) in 2017. All strains were identified by matrix-assisted laser desorption/ionization time-of-flight mass spectrometry (MALDI-TOF MS) using the VITEK Mass Spectrometer (BioMerieux, Lyons, France). All strains were stored at −80°C and incubated on blood agar plates at 37°C for 18–24 h before use.

### Antimicrobial Susceptibility Testing

Antimicrobial susceptibility testing was undertaken by the agar dilution method according to the latest (Clinical and Laboratory Standards Institute, 2020). Briefly, an overnight cultured single colony was suspended in sterile NaCl (0.85%), and the suspensions were adjusted to the turbidity equal to 0.5 McFarland standard (1.5 × 10^8^ CFU/mL). Then, the mixture was further diluted to 1:10 and evenly spotted onto the drug-containing MH agar plate; the results were observed after incubation at 37°C for 16–18 h. CDB (lot number: Z20O9B72911, Shanghai Yuanye Biotechnology Co., Ltd., China) was dissolved in dimethyl sulfoxide (DMSO) and tested over a range of 1–512 μg/mL. *S. aureus* ATCC 29213 was employed for the quality control of the strain. The minimal inhibitory concentration (MIC) values were tested in 3 independent experiments.

### Bacterial Growth Curve Assay

The bacterial growth curve was determined as previously described, with minor modifications (Zhou et al., 2018). Briefly, the 6 clinical *S. aureus* strains (JP-2541, JP-2718, JP-2744, JP-2850, JP-2918, and JP-3053) were isolated from patients diagnosed with different types of infected wound, as details in Supplementary Table 1. *S. aureus* ATCC 29213 served as the quality control strain. The 7 isolates mentioned above were cultured in fresh Luria–Bertani (LB) broth at 37°C with shaking at 180 revolutions per minute (rpm) to obtain an OD_600_ value of 0.3, followed by further dilution to 1:100 with 20 mL of fresh LB broth in the presence of CDB (16, 32, 64, 128, and 256 μg/mL, respectively) as well as diluted 1:100 with fresh LB broth alone as the control group, followed subsequently by incubation at 37°C at 180 rpm for overnight. The OD_600_ value was measured every hour for 24 h. Therefore, a total of 24 points of data of each sample were collected. All experiments were independently repeated in triplicate.

### Time-Kill Curve Assay

The time-kill curve assay for 7 *S. aureus* strains (JP-2541, JP-2718, JP-2744, JP-2850, JP-2918, JP-3053, and ATCC 29213) was performed using a previously standardized method (Foerster et al., 2015, 2016). Briefly, overnight cultures were diluted in 20 mL of fresh LB broth to a final concentration of approximately 1 × 10^7^ CFU/mL, and CDB at 0, 1/2 × MIC, 1 × MIC, 2 × MIC concentrations were added, respectively. Finally, viable colony counts were determined at 0, 2, 4, 6, 12, and 24 h after incubation at 37°C at 180 rpm. The time-kill curves of each strain were plotted with the number of bacteria per mL (CFU/mL) with the ordinates and time (h) as the abscissas.

### Crystal Violet Biofilm Assay

The biofilm formation ability assay was performed according to the methods by O’Toole with some minor modifications (Niemirowicz et al., 2016). An overnight culture of each isolate was incubated at 37°C/180 rpm up to the logarithmic phase with an OD_600_ value of 0.6, and the turbidity was adjusted to 0.5 McFarland standard, further diluted to 1:100, and CDB at 0, 32, 64, 128, 256, and 512 μg/mL concentrations were added, respectively. Then, 100 μL of the dilution was added to the 96-well polystyrene micro-test plate (Flat bottom with lid, Sterile; Corning, United States), and 3 replicate wells were set up. Following 24-h incubation at 37°C with shaking at 75 rpm, the upper planktonic bacteria was decanted, and biofilms attached to the well surfaces were stained with 100 μL of 1% (*w*/*v*) crystal violet solution (lot number: NO.20190324, Beijing Solarbio Biotechnology Co., Ltd., China) for 15 min. The bound dye was solubilized for 30 min with 100 μL of the eluent (95% absolute ethanol and 5% glacial acetic acid) and subsequently quantified by measuring the OD_595_ value by the Multiskan FC Microplate Reader.

### Scanning Electron Microscope (SEM) Analysis

In addition to measuring the effects of CDB against *S. aureus*, SEM observation was performed, with some minor modifications (Jiamboonsri et al., 2011; Singh et al., 2017). Briefly, the prepared inoculum (300 μL) of JP-2541 and *S. aureus* ATCC 29213 was transferred into LB broth (2.7 mL) in the presence and absence of 1 × MIC CDB, the positively charged glass slide was placed into each well and subsequently incubated at 37°C overnight. The bacterial cells on the coverslip were fixed in 2.5% (*w*/*v*) of glutaraldehyde at 4°C for 4 h and rinsed with 0.1 M of phosphate buffer (pH 7.2), followed by dehydration in graded ethanol (30, 70, and 100%) and drying at the room temperature for overnight. Finally, the dried samples were covered with gold and observed under the S-3000N scanning electron microscope (SEM) (Hitachi, Japan) at various levels of magnification.

### Cell Membrane Tests

Actively growing *S. aureus* culture of each isolate was treated with serial concentrations of CDB (0, 32, 64, 128, 256, and 512 μg/mL) at 37°C for 6 h. The alkaline phosphatase release levels of bacterial cell membrane disruption of each isolate were assessed by using a commercial kit (Solarbio, Beijing, China) (Qu et al., 2019). In an alkaline environment, AKP/ALP catalyzes the formation of phenyl disodium phosphate into free phenols. Phenols react with potassium ferricyanide and 4-aminoantipyrine to form red quinone compounds with characteristic absorbance at 510 nm. The activity of CDB against *S. aureus* cell membrane was calculated by measuring the absorbance increase rate at 510 nm.

### Quantitative Real-Time PCR (qRT-PCR)

All *S. aureus* strains (JP-2541, JP-2718, JP-2744, JP-2850, JP-2918, JP-3053, and ATCC 29213) were treated with or without 1/4 × MIC CDB, after which the total RNA of *S. aureus* strains were extracted from the bacterial culture using the Bacterial RNA Miniprep Kit (Biomiga, Shanghai, China). The cDNA was reversed with 1000-ng RNA templates using the RevertAid First Strand cDNA Synthesis Kit (Thermo Scientific, Waltham, MA, United States). These primers are listed in Table 1 (Duan et al., 2018). The expression levels of alpha-hemolysin gene (*hla*), response regulator gene (*saeR*), and histidine kinase gene (*saeS*) were analyzed by qRT-PCR. *gyrb* was used as an internal reference. As previously described, qRT-PCR was performed using the TB Green Premix Ex Taq II (Tli RNase H Plus) (2×) (Takara, Japan) (Xu et al., 2020).

**TABLE 1:** Primers used for qRT-PCR.

Primer name	Sequence (5′→3′)
*gyrb*-RT-F	ACATTACAGCAGCGTATTAG
*gyrb*-RT-R	CTCATAGTGATAGGAGTCTTCT
*hla*-RT-F	TGGTAATCATCACGAACTC
*hla*-RT-R	GCAGCAGATAACTTCCTT
*saeR*-RT-F	GTCGTAACCATTAACTTCTG
*saeR*-RT-R	ATCGTGGATGATGAACAA
*saeS*-RT-F	TGTATTTAAAGTGATAATATGAGTC
*saeS*-RT-R	CTTAGCCCATGATTTAAAAACACC

### Statistical Analysis

The GraphPad Prism, version 8.02 (GraphPad Software, San Diego, CA, United States) was used for data analysis. The results were expressed as means ± SD, and comparison among the studied groups was conducted by the Student’s *t*-test. Significance was considered at *P* < 0.05, and all tests were two-tailed.

## Results

### Determination of MICs of CDB

Antimicrobial susceptibility testing revealed that the MIC values of CDB against *S. aureus* ranged from 32 to 128 μg/mL by the agar dilution method (Table 2).

**TABLE 2:** Antibiotic susceptibility of CDB against 46 clinical *S. aureus* isolates and *S. aureus* ATCC 29213.

Strain	CDB MIC (μg/mL)	Strain	CDB MIC (μg/mL)
JP-2433	64	JP-2743	64
JP-2437	64	JP-2744	64
JP-2476	64	JP-2764	64
JP-2509	64	JP-2768	64
JP-2541	64	JP-2786	64
JP-2560	64	JP-2800	64
JP-2568	64	JP-2826	64
JP-2582	64	JP-2831	64
JP-2590	128	JP-2842	64
JP-2608	64	JP-2850	64
JP-2611	64	JP-2890	64
JP-2617	64	JP-2902	32
JP-2626	64	JP-2910	64
JP-2628	64	JP-2918	64
JP-2630	64	JP-2928	64
JP-2631	64	JP-2942	64
JP-2632	128	JP-2957	64
JP-2644	64	JP-2975	64
JP-2674	64	JP-3019	64
JP-2679	64	JP-3023	64
JP-2694	64	JP-3053	64
JP-2718	64	JP-3058	64
JP-2730	64	ATCC 29213	64
JP-2738	64		

### Bacterial Growth Curve Assay

**FIGURE 1:**
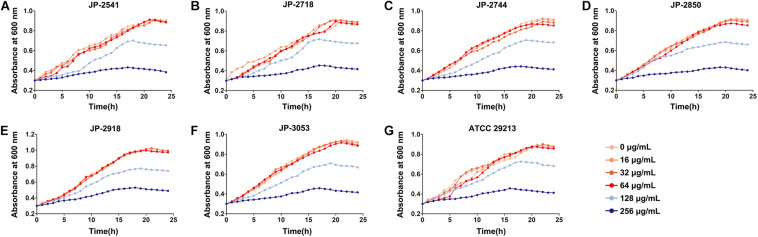
Effects of different concentrations of CDB on the growth of *Staphylococcus aureus.* **(A)** growth curves for JP-2541; **(B)** growth curves for JP-2718; **(C)** growth curves for JP-2744; **(D)** growth curves for JP-2850; **(E)** growth curves for JP-2918; **(F)** growth curves for JP-3053; and **(G)** growth curves for *S. aureus* ATCC 29213.

By measuring the effects of CDB with different concentrations on the growth of *S. aureus*, it was found that CDB had no effect on the growth of *S. aureus* at the concentration ≤64 μg/mL when compared with the control group; while CDB with a concentration ≥128 μg/mL could effectively inhibit the growth of *S. aureus* (Figure 1).

### Time-Kill Curve Assay

**FIGURE 2:**
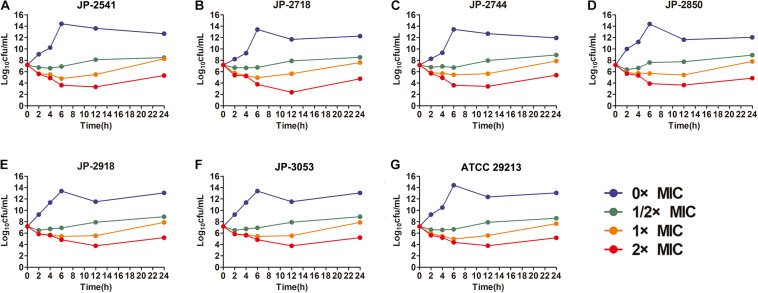
Time-kill curves of different concentrations of CDB against *Staphylococcus aureus*. **(A)** time-kill curves for JP-2541; **(B)** time-kill curves for JP-2718; **(C)** time-kill curves for JP-2744; **(D)** time-kill curves for JP-2850; **(E)** time-kill curves for JP-2918; **(F)** time-kill curves for JP-3053; and **(G)** time-kill curves for *S. aureus* ATCC 29213.

The time−kill curve assay was performed for 6 clinical *S. aureus* isolates (JP-2541, JP-2718, JP-2744, JP-2850, JP-2918, and JP-3053) and *S. aureus* ATCC 29213. At 0–12 h, our results demonstrated that all isolates kept growing in the absence of CDB and that all these strains remained at the initial inoculation level at the 1/2 × MIC CDB. Interestingly, at 0–12 h, when compared with the initial inoculum, the colony counts of the bacteria decreased by approximately 100 times at the 1 × MIC CDB and were maintained at the level of the bacteria; while the growth of all strains was inhibited at 2 × MIC CDB. After 12 h, the results of this experiment revealed that *S. aureus* demonstrated a remarkable trend of increasing growth at all concentrations of CDB (Figure 2).

### Efficacy of CDB on Biofilm Formation of *S. aureus*

**FIGURE 3:**
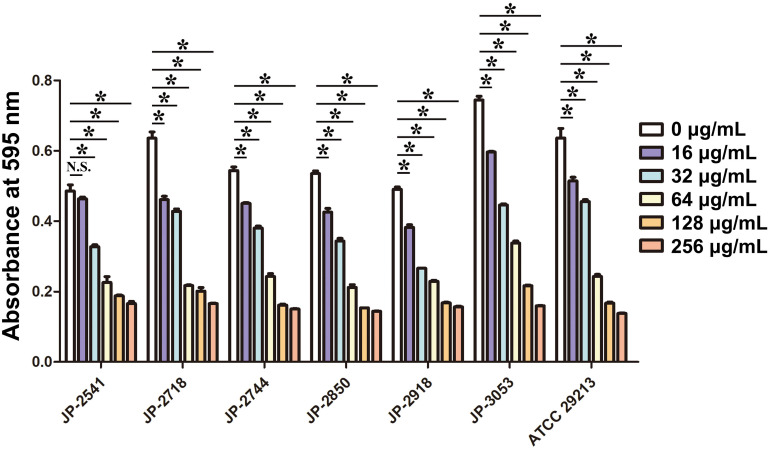
Effects of different concentrations of CDB on the biofilm formation ability of *Staphylococcus aureus*. *P < 0.05; N.S., *P* > 0.05.

The biofilm formation ability of *S. aureus* in the LB broth with different concentrations of CDB was compared. Our result illustrated biofilm structures of *S. aureus* in the LB broth in the presence and absence of CDB and noted that the differences in the biofilm formation ability between the drug-containing LB broth group and LB broth group be statistically significant (*P* < 0.05; Figure 3).

### Scanning Electron Microscope (SEM) Analysis

**FIGURE 4:**
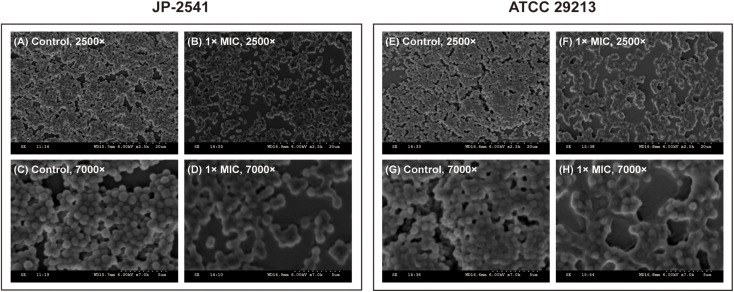
Scanning electron microscope (SEM) images of JP-2541 and *S. aureus* ATCC 29213 after treatment with CDB. **(A)** control group of JP-2541, 2,500 × magnification; **(B)** 1 × MIC CDB against JP-2541, 2,500 × magnification; **(C)** control group of JP-2541, 7,000 × magnification; **(D)** 1 × MIC CDB against JP-2541, 7,000 × magnification; **(E)** control group of *S. aureus* ATCC 29213, 2,500 × magnification; **(F)** 1 × MIC CDB against *S. aureus* ATCC 29213, 2,500 × magnification; **(G)** control group of *S. aureus* ATCC 29213, 7,000 × magnification; and **(H)** 1 × MIC CDB against *S. aureus* ATCC 29213, 7,000 × magnification.

The visualization of JP-2541 and *S. aureus* ATCC 29213 cell morphology through SEM after treatment with CDB at 1 × MIC concentration and control cells are presented in Figure 4. The surface of *S. aureus* cells untreated with CDB (control group) formed a thick biofilm composed of aggregates and microcolonies on the coverslip at 2500 × magnification, and the cell morphology was observed to be smooth with some typical characters of ball shape (Figures 4A,E). However, *S. aureus* cells treated with 1 × MIC CDB appeared sparse and dispersed, and the number of cells was significantly reduced (Figures 4B,F). Moreover, there was no change in the morphology of the control cells at 7000 × magnification (Figures 4C,G), whereas the CDB-treated cells showed partial destruction (Figures 4D,H).

### Cell Membrane Tests

**FIGURE 5:**
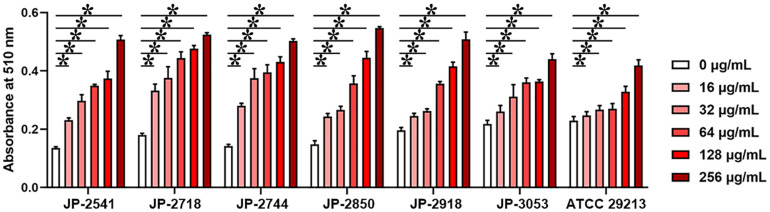
Different concentrations of CDB used to disrupt the bacterial cell membrane integrity. *P < 0.05; N.S., *P* > 0.05.

On treating these 7 isolates with different concentrations of CDB, the differences in the release levels of alkaline phosphatase between the treated and control groups were statistically significant (*P* < 0.05; Figure 5). Together, these data indicated that CDB could lead to increased permeability and the weakening of the cell membrane of each isolate.

### qRT-PCR

**FIGURE 6:**
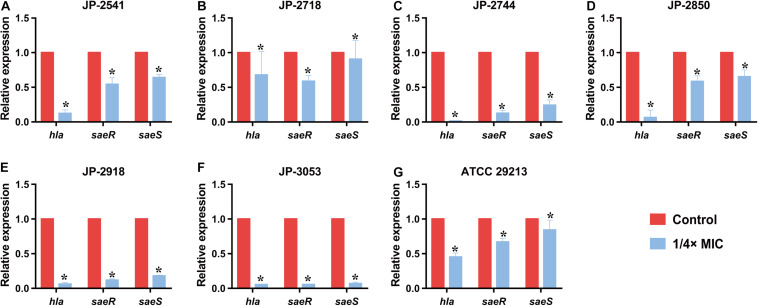
Relative expression level of *hla*, *saeR*, and *saeS* in *S. aureus* strains after culturing with 1/4 × MIC concentration of CDB. *P < 0.05. **(A)** Genes expression level in JP-2541. **(B)** Genes expression level in JP-2718. **(C)** Genes expression level in JP-2744. **(D)** Genes expression level in JP-2850. **(E)** Genes expression level in JP-2918. **(F)** Genes expression level in JP-3053. **(G)** Genes expression level in *S. aureus* ATCC 29213.

The results of qRT-PCR revealed that the relative expression levels of *hla* and *saeRS* in these strains were downregulated significantly after the exposure of the 1/4 × MIC concentration of CDB (*P* < 0.05; Figures 6A–G). These results indicated that CDB could decrease the capacity of alpha-hemolysin production of *S. aureus* strains through the inhibition of the expression of *hla*, *saeR*, and *saeS*.

## Discussion

The skin serves as a protective physical barrier against invading microbes, including pathogens (Jun et al., 2015; Chaudhary et al., 2019). Wounds are inevitably infected by microorganisms in nature during the formation and healing processes. Infected wounds not only prolong the healing time but also threaten the lives of the patients at times (Meara et al., 2015). The pathogens responsible for the infection vary in different wound environments; nonetheless, *S. aureus* is the most common pathogen causing infected wounds (Alves et al., 2018; Rashid et al., 2019). In this scenario, it has been observed that CDB offers potential health benefits and could be exploited for treating infected wounds in clinical settings (Wan et al., 2019; Lin et al., 2020). Thus, we investigated the underlying antibacterial activities of CDB against *S. aureus* isolated from wound specimens and provided scientific evidence for antibiotic treatment of infected wounds with CDB.

To the best of our knowledge, the present study is the first to shed light on the antibacterial and anti-biofilm efficacy of CDB against *S. aureus*. Based on the results of bacterial growth curve assay and time-kill curve assay, we concluded that CDB could inhibit the growth of *S. aureus* in a concentration-dependent manner. Meanwhile, sub-inhibitory concentrations of CDB could reduce the biofilm formation ability of *S. aureus* and disrupt its cellular membrane. The above findings were further supported by SEM findings. We further uncovered that CDB could reduce the virulence factors of *S. aureus* by downregulating the expression levels of *saeR*, *saeS*, and *hla* and inhibiting the hemolytic activity.

CDB is mainly distributed in Hainan, Guangxi, and Southern Yunnan in China, and it is produced from *Dracaena cochinchinensis* in Indonesia, Australia, Africa, and other countries. In recent years, owing to the increasing scope of clinical application of CDB, many scholars and clinicians have conducted in-depth research on its chemical composition and pharmacological actions. Previous studies have revealed that the main components of CDB are phenolics and flavonoids (Gupta and Gupta, 2011; Stefano et al., 2014; Al-Fatimi, 2018). Moreover, saponins, terpenoids, resveratrol, and other ingredients are present in it. CDB has antibacterial, anti-inflammatory, analgesic, and anti-platelet aggregation properties and is capable of promoting blood circulation and epidermal repair, besides displaying other pharmacological effects (Luo et al., 2011; Liu et al., 2013). However, little is known about the underlying antibacterial activities of CBD against *S. aureus*. Some studies have confirmed that CBD is rich in proanthocyanidins, phenolics, and flavonoids, which constitute 90% of its dry weight (Jones, 2003; Al-Fatimi, 2018; Pona et al., 2019). Proanthocyanidin, as a final product of the flavonoid biosynthetic pathway, is known to effectively prevent and cure bacterial infections (Rauf et al., 2019). Also, these phenolic compounds can be easily degraded (Escobar et al., 2018). As shown in Figure 2, *S. aureus* exhibited remarkably increased cell growth at all concentrations of CDB after treatment for 12 h, which is consistent with previous reports. This phenomenon reminds us that we should pay attention to such time-dependent characteristics when using CDB in clinical settings.

Previous studies have confirmed that in the environment of chronic infected wounds, the formation of bacterial biofilms could make the bacteria more adaptable to the external environment by increasing the adhesion to the wound surface and evading the host’s immune function (Krychowiak et al., 2014). In addition, the biofilm structure could significantly enhance the pathogen’s resistance to antibiotics by preventing the drugs from entering the bacterial cell, which is the main reason for persistent inflammation of the wound and difficulty in healing (van Wamel, 2017). Therefore, it is essential to find compounds that could inhibit the formation of biofilms. In our study, crystal violet biofilm assay and SEM analysis revealed that sub-inhibitory concentrations of CDB could effectively lower the biofilm formation ability of *S. aureus*. Biofilm formation of *S. aureus* is an important factor that determines the wound healing process and patient mortality (Bhattacharya et al., 2015; Roy et al., 2020). Besides, Tsung-Jung Ho et al. confirmed that CDB could stimulate angiogenesis and promote cell proliferation and migration (Ho et al., 2016). We speculated that these might be the important reasons for CDB promoting the wound healing process.

In addition to biofilm formation, virulence factors of *S. aureus* play crucial roles in wound healing either directly or indirectly. Among the various virulence determinants, alpha-hemolysin (Hla) is one of the most significant virulence factors in *S. aureus* wound infections, which results in attenuated production by inhibiting the expression level of the gene encoding Hla (*hla*) or global regulatory genes such as *saeS* and *saeR* (Duan et al., 2018; Gudeta et al., 2019; Putra et al., 2019). The *saePQRS* system is a global regulator of *S. aureus*, and among them, *saeS* (encoding a histidine kinase) and *saeR* (encoding a response regulator) play regulatory roles in controlling the expression of *hla* (Gudeta et al., 2019; DelMain et al., 2020). As shown in qRT-PCR results, upon comparing the gene expression data of the control group with that of the group treated with 1/4 × MIC of CDB, the transcription levels of *saeR*, *saeS*, and *hla* were downregulated. Our results revealed that CDB could decrease Hla production by *S. aureus* owing to a reduction in the expression of *saeR*, *saeS*, and *hla*, thereby potentially weakening the virulence determinants of the pathogen.

Therefore, we next sought to further investigate the activities of CDB against *S. aureus in vivo* by constructing the mouse model of *S. aureus* skin infected wound, and then visual observation of surface healing, bacterial counts, histology observation, and immunohistochemical analysis can be performed in the uninfected- and infected-wound groups. Most importantly, we are aware that understanding the underlying antibacterial activities of CDB against *S. aureus* is the crucial first step in exploring the molecular mechanisms or specific pathways of CDB to curb the growth, biofilm formation, and virulence factors of the pathogen.

## Conclusion

In conclusion, CDB, which is one of the most precious traditional Chinese medicine, exerts positive antibacterial efficacy on *S. aureus*, and can also reduce the biofilm formation and retard the virulence factors alpha-hemolysin of *S. aureus* by downregulating the expression levels of *saeR*, *saeS*, and *hla* genes. Our study provides new insights into the rational use of CDB for the treatment of infected wounds caused by *S. aureus* for the first time. These findings together indicate that CDB possesses significant potential as an alternative for the treatment of infected wounds caused by *S. aureus* in clinical settings. Furthermore, it will be worthwhile to further investigate the activities of CDB against *S. aureus in vivo*.

## Data Availability Statement

The raw data supporting the conclusions of this article will be made available by the authors, without undue reservation.

## Ethics Statement

The studies involving human participants were reviewed and approved by The Ethics Committee of the First Affiliated Hospital of Wenzhou Medical University (approval number: 2021–R003). Written informed consent for participation was not required for this study in accordance with the national legislation and the institutional requirements.

## Author Contributions

XZ and LC conducted the experiments, analyzed the data, and wrote the manuscript. WZ participated in the experiments and writing. WL, ZW, XT, and RF analyzed the data. XZ and YS supervised the manuscript. TZ designed the study. All authors read and approved the final version of the manuscript for submission.

## Conflict of Interest

The authors declare that the research was conducted in the absence of any commercial or financial relationships that could be construed as a potential conflict of interest.

## Supplementary Material

Supplementary Table 1 Patient clinical data and characteristics of 6 clinical *S. aureus* isolates.
