# Supplementary material for: Antibacterial and Anti-biofilm Efficacy of Chinese Dragon’s Blood Against Staphylococcus aureus Isolated From Infected Wounds
Source: Front Microbiol. 2021 Jun 4;12:672943. doi: 10.3389/fmicb.2021.672943 (PMC8213214; doi:10.3389/fmicb.2021.672943)
Supplement: Supplementary Table 1 — Patient clinical data and characteristics of 6 clinical S. aureus isolates. [file Table_1.DOCX]

**Table S1 Patient clinical data and characteristics of 6 clinical *S. aureus* isolates**

| Strain | Isolation date | Specimen | Gender^a^ | Age | Ward | Discharge Diagnosis |
| --- | --- | --- | --- | --- | --- | --- |
| JP-2541 | 20/03/2017 | Wound | M | 71 | Endocrinology | Diabetic Foot Ulcer |
| JP-2718 | 11/07/2017 | Wound | M | 70 | Dermatology | Drug-induced dermatitis with infection |
| JP-2744 | 27/07/2017 | Wound | M | 66 | Urology | Decubitus ulcer |
| JP-2850 | 20/09/2017 | Wound | M | 45 | Endocrinology | Carbuncle (right ear) |
| JP-2918 | 18/10/2017 | Wound | M | 64 | Hand Surgery | Skin ulcer (right hand) |
| JP-3053 | 17/12/2017 | Wound | F | 15 | Burn Surgery | Burn (right foot) |

^a^ *M, Male*; *F, Female*.
